# Spine Injuries Related to Ocean Waves: Case Series of Unusual Injuries

**DOI:** 10.7759/cureus.3335

**Published:** 2018-09-19

**Authors:** Luis A Robles

**Affiliations:** 1 Neurosurgery, CMQ Premier Hospital, Puerto Vallarta, MEX

**Keywords:** spine injuries due to ocean waves, cervical injuries in the ocean, ocean waves injuries

## Abstract

Spine injuries related to ocean wave accidents are little known because they exclusively occur in beach destinations. This unusual type of trauma happens when these patients are slammed or caught by a wave, rolled over, and driven into the water, usually hitting the head on the seabed. Due to the kinematics of ocean wave accidents, spine injuries occur almost exclusively in the subaxial cervical spine. Several cases of spine injuries associated with ocean wave accidents are described. These ocean bathers presented with spinal trauma in levels other than the subaxial cervical spine. Two patients presented with injuries in the upper cervical spine, another patient showed a thoracic fracture, and finally, another patient sustained a lumbar compression fracture. All patients were tourists with minimal experience with swimming in the ocean who very likely ignored the danger of waves. This study shows that spine injuries related to ocean waves can occur at any level of the spine. Therefore, emergency physicians and spine surgeons must be aware of unusual locations of this unique type of trauma.

## Introduction

Although people feel safe in the shallow water, a variety of injuries related to ocean waves can occur, including spinal injuries [1-6]. Previous studies reporting patients with spinal injuries related to ocean waves showed that most injuries occur in the subaxial cervical spine [5,7]. In this paper, the author presents a case series of patients who experienced spine injuries in areas different from the subaxial spine, thus showing that spine injuries related to ocean waves may occur at any area of the spine. All patients in this study were initially assessed in the emergency room and were treated accordingly by the author.

## Case presentation

Case series

Case 1

This is a 62-year-old male tourist who was playing in the ocean waves. He was caught and rolled over by a wave and was driven into the water, and he hit the head over the seabed. He experienced transient numbness and weakness in the four limbs. The patient reported only neck pain; on examination, bruises were observed in the left frontotemporal area, and he had normal motor and sensory function of the upper and lower limbs.

**Figure 1:**
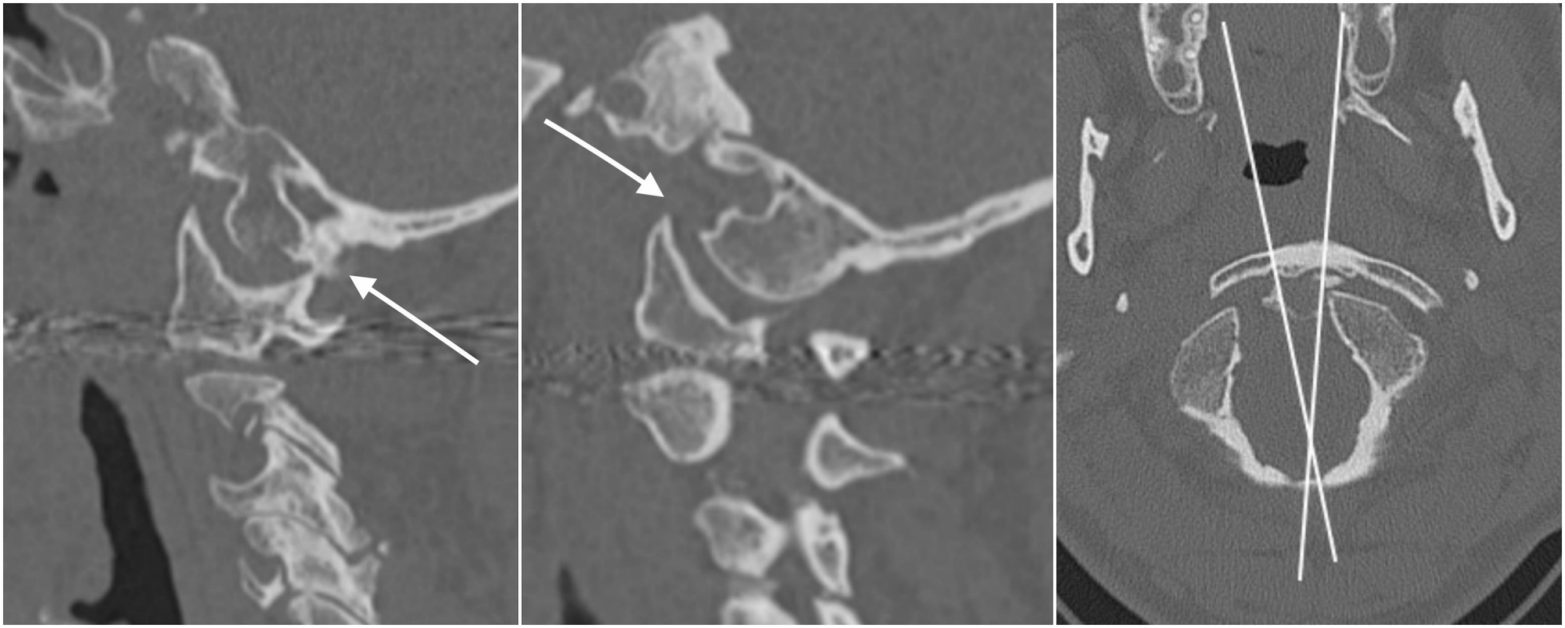
Computed tomography (CT) scan, atlanto-occipital rotatory dislocation Left, right parasagittal CT scan showing a widened condylar-C1 interval (2.5 mm) and a condylar fracture (arrow). Center, left parasagittal CT scan displaying widening of the condylar-C1 interval (4.3 mm). Right, axial CT scan showing rotatory displacement to the right of the occipital condyles over the atlas. The rotational angle is 13°.

Radiological tests showed an atlanto-occipital dislocation and other traumatic injuries. Conventional X-rays were normal. Axial computed tomography (CT) scan showed an atlanto-occipital rotatory dislocation characterized by rotatory displacement of the atlanto-occipital joints into the right; parasagittal views showed widening of the condylar-C1 interval in both sides (right 2.5 mm, left 4.3 mm) indicating disruption of these joints. In additon, a right occipital condyle fracture was observed (Figure 1).

Magnetic resonance imaging (MRI) showed distraction injury of atlanto-occipital and atlanto-axial articular capsules; in addition, the right alar ligament was attached to the bone fragment of the condylar fracture. Because the patient was a tourist, he was transferred to his country of origin to continue treatment. He was treated with occipitocervical fixation. 

Case 2

**Figure 2:**
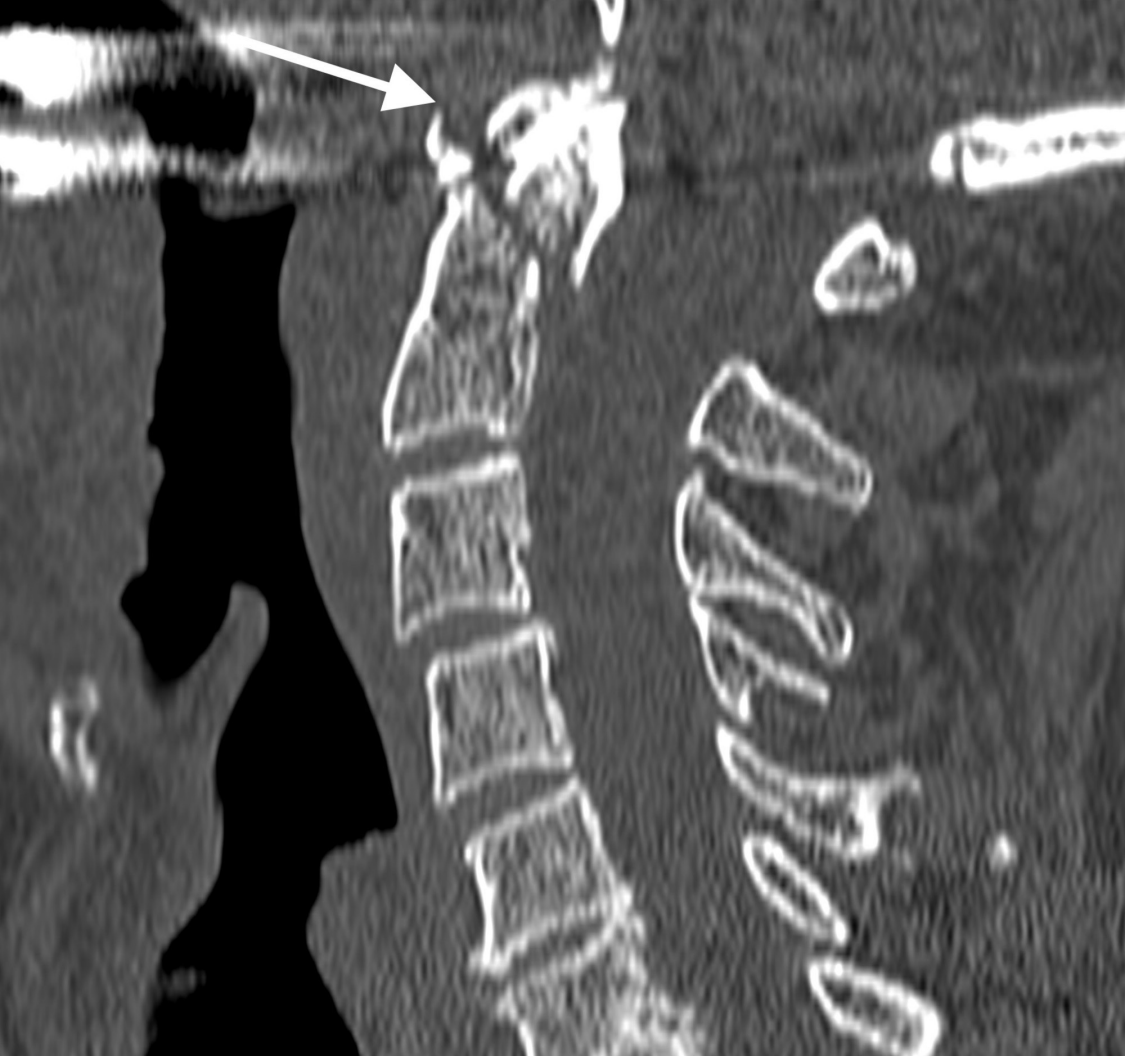
Computed tomography (CT) scan, odontoid fracture Sagittal CT scan showing a type II odontoid fracture. A 3 mm displacement is observed (arrow).

This 71-year-old male tourist was swimming in the ocean close to the shore when he was rolled over by a wave. He was driven into the water and hit the head on the sea bottom. He immediately experienced tingling in the four limbs that reversed spontaneously. At admission, he only reported neck pain. On examination, he was neurologically intact. Some bruises were observed on the forehead. Radiological tests showed a type II odontoid fracture with displacement of 3 mm (Figure 2).

The patient was treated with immobilization. He traveled to his country of origin to continue medical treatment.

Case 3

**Figure 3:**
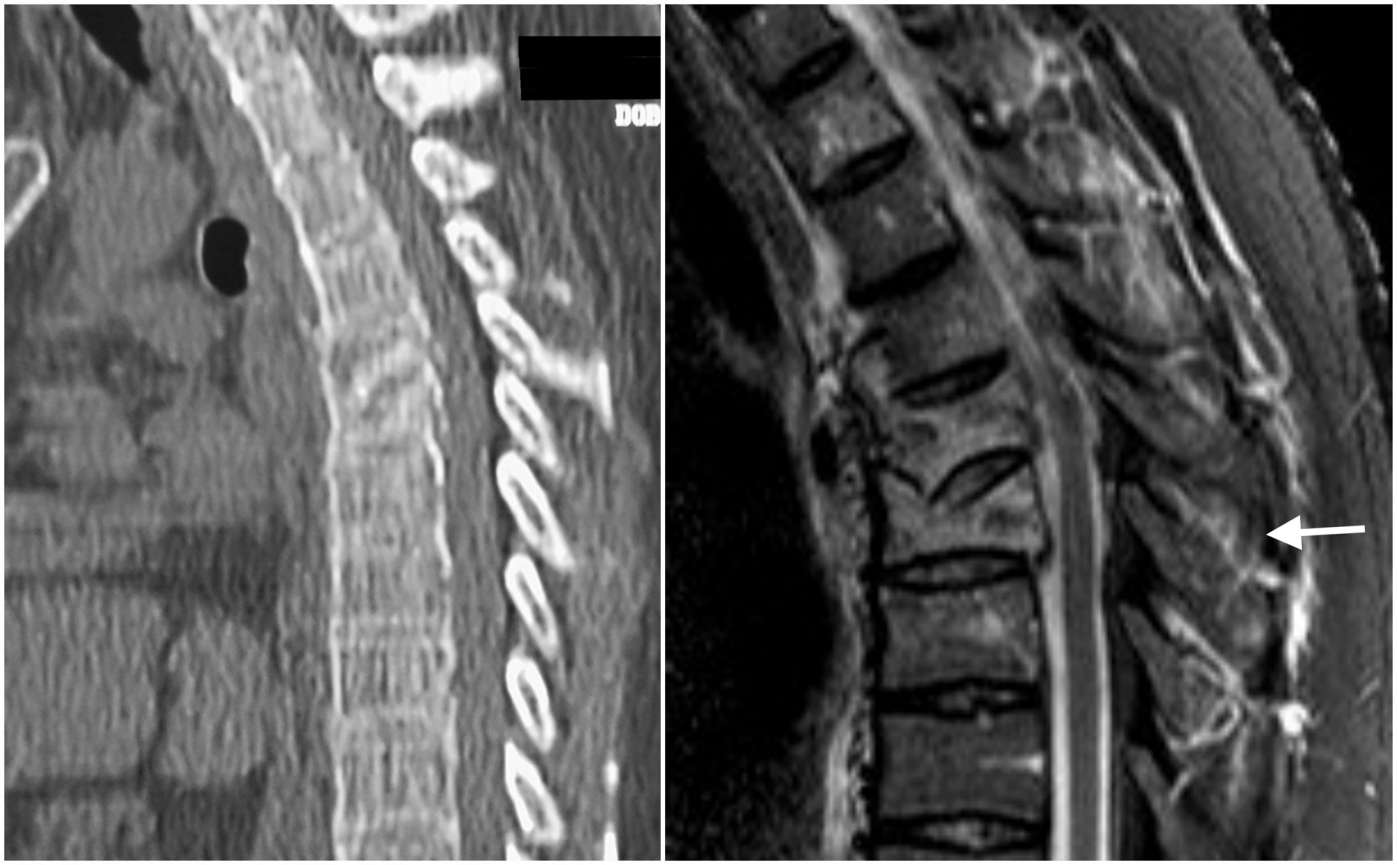
Thoracic fracture Left, sagittal computed tomography (CT) scan showing compression fracture of T4-5 vertebral bodies. Right, sagittal magnetic resonance imaging (MRI) STIR showing the fractures and changes in the intensity of vertebral bodies. In addition, injury to interspinous ligaments is observed (arrow). STIR - Short-TI Inversion Recovery.

A 61-year-old male tourist was swimming in the ocean when he was caught by a big wave. He initially hit the sea bottom with his face and was violently rolled over. At admission, he referred intense thoracic pain. On examination, severe contusion and ecchymosis were observed on the right orbital area causing complete eye occlusion. A very painful area was identified in the upper spinal thoracic area; he referred intense pain even with mild movements. He was neurologically intact. A CT scan and MRI showed compression fractures at T4-T5, the canal spinal was preserved, and the MRI showed disruption of the posterior ligamentous complex (Figure 3).

Instrumentation from T3 to T6 and fusion were performed. The patient achieved a good outcome, he did not show any neurological deterioration or complications.

Case 4

**Figure 4:**
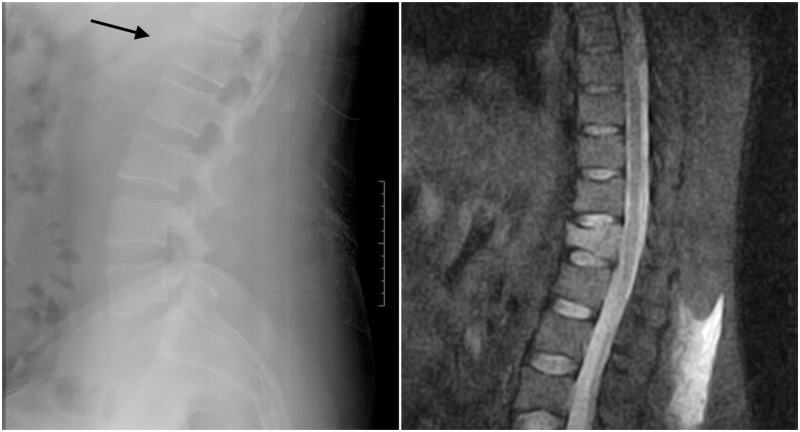
Lumbar compression fracture Left, lateral radiograph showing an L1 compression fracture (arrow). Right, magnetic resonance imaging (MRI) STIR displaying hyperintensity of the L1 vertebral body indicating an acute compression fracture. STIR - Short-TI Inversion Recovery.

A 40-year-old male tourist was swimming in the ocean surf zone when he was caught and rolled over by a wave, and he landed on his buttocks over the seabed. At admission, he referred intense low back pain. Neurologically he was intact. Radiological tests showed a wedge-compression fracture located at L1 (Figure 4).

The patient was treated conservatively with analgesics and was discharged with a brace.

## Discussion

Injuries to beachgoers caused by ocean waves are more common and severe than expected. These injuries usually occur in shallow water close to the shore where many people play in the waves. Frequently, spine injuries occur in people who are slammed or caught and rolled over by ocean waves when they are practicing different activities in the surf zone. These patients are violently driven into the water and land with the head on the seabed; for this reason, the most common type of spine trauma occurs in the cervical spine. These accidents generally occur in tourists because they probably underestimate or ignore the power of the ocean, or they don't know how to swim in ocean waves. This situation can be corroborated by the fact that beaches in this resort are also frequently visited by locals; and until now, I have not treated a local resident with this type of injury.

Most of the spine injuries related to ocean waves are observed in the subaxial cervical spine [1,5]. Robles reported 16 cases of ocean bathers sustaining spine injuries when they were rolled over by ocean waves, and all of these injuries were located in the subaxial cervical spine [5]. In that study, most cases were older patients who presented with central cord syndrome associated with preexisting cervical spondylosis. The most common mechanism of injury was hyperextension, which was observed in 75% of cases.

Chang et al. reported a study of patients experiencing cervical spine injuries related to different water sport activities in the ocean; most of these patients had injuries in the subaxial cervical spine, C5 and C6 being the most commonly injured levels [7].

Cheng et al. reported 14 cases of cervical spine injuries caused by bodysurfing accidents [1]. Most of these injuries were observed in the subaxial cervical spine. In this study, injuries occurred in younger patients, and seemingly the most frequent mechanism of injury was hyperflexion.

In cervical spine injuries, the posture of the head and neck at the time of the trauma and the location and direction of the force vector will dictate the pattern of spinal injury; the kinetic energy imparted predominantly dictates the magnitude of the damage [8]. The same statement applies in patients with spine injuries secondary to ocean waves. The kinematics of trauma in these patients usually leads to the occurrence of cervical spine injuries; however, as demonstrated in this paper, the position of the spine at the moment of the accident will dictate the level of the resulting spinal injury. The fact that patients are rolled over by the wave suggests that any part of the spine is exposed to these injuries. The present study reports injuries in different levels of the spine including the craniocervical junction, thoracic and lumbar areas.

Different mechanisms of injuries were observed in this case series: 1) in the first case, the facial injuries and radiological findings suggest that the mechanism of injury was lateral flexion of the head. Cases of atlanto-occipital dislocation usually show a complete disruption of all stabilizing ligaments of the craniocervical junction, leading to high mortality. In this case, the loss of the stability was incomplete, so the dislocation was less severe. This scenario and the presence of a wide spinal canal at this level led to a milder injury; 2) In case 2, the facial trauma location and radiological findings suggest that the mechanism of injury in this odontoid fracture was hyperextension; 3) In case 3, imaging indicates that the mechanism of injury was flexion-distraction of the thoracic spine, causing several compression fractures associated to disruption of the posterior ligamentous complex; 4) Finally, in case 4, the kinematics of trauma and radiological findings indicate that the mechanism of injury was axial compression of the lumbar spine.

Ocean waves can impart a lot of energy and it is even considered that being hit by ocean waves may have the same effect as being hit by a compact car. This situation was corroborated in the case of the atlanto-occipital dislocation because it is known that atlanto-occipital injuries occur secondary to very high energy accidents.

Although severe neurological impairment has been reported in these type of accidents, in this study, all patients were neurologically intact. The good neurological outcome was achieved because none of these patients had anatomically significant compromise of the spinal canal.

Most injuries in this case series occurred in older patients. This scenario could be explained by the fact that an older person has less physical ability to react or respond to accidents.

## Conclusions

Spine injuries related to ocean waves very often occur in tourists. Although spine trauma in these patients is very commonly seen in the subaxial cervical spine, this study shows that spine injuries can occur at any level of the spine. Emergency physicians and spine surgeons, especially those who practice in beach destinations, should be aware of unusual locations of this type of spine trauma.
